# Repetitive binge-like consumption based on the Drinking-in-the-Dark model alters the microglial population in the mouse hippocampus

**DOI:** 10.31083/j.jin2004094

**Published:** 2021-12-30

**Authors:** James C. Nelson, Eva Greengrove, Kala N. Nwachukwu, Isabella R. Grifasi, S. Alex Marshall

**Affiliations:** 1 Department of Biological & Biomedical Sciences, North Carolina Central University, Durham, NC 27707, USA; 2 Department of Basic Pharmaceutical Sciences, Fred P. Wilson School of Pharmacy, High Point University, High Point, NC 27268, USA

**Keywords:** Alcohol use disorder, Microglia, Neuroimmune, Amygdala, Hippocampus, Binge-like drinking

## Abstract

Alcoholism causes various maladaptations in the central nervous system, including the neuroimmune system. Studies of alcohol-induced dysregulation of the neuroimmune system generally focus on alcohol dependence and brain damage, but our previous research indicates that repetitive binge-like consumption perturbs cytokines independent of cell death. This paper extends that research by examining the impact of binge-like consumption on microglia in the hippocampus and the amygdala. Microglia were assessed using immunohistochemistry following binge-like ethanol consumption based on Drinking-in-the-Dark model. Immunohistochemistry results showed that binge-like ethanol consumption caused an increase in Iba-1 immunoreactivity and the number of Iba-1+ cells after one Drinking-in-the-Dark cycle. However, after three Drinking-in-the-Darkcycles, the number of microglia decreased in the hippocampus. We showed that in the dentate gyrus, the average immunoreactivity/cell was increased following ethanol exposure despite the decrease in number after three cycles. Likewise, Ox-42, an indicator of microglia activation, was upregulated after ethanol consumption. No significant effects on microglia number or immunoreactivity (Iba-1 nor Ox-42) were observed in the amygdala. Finally, ethanol caused an increase in the expression of the microglial gene Aif-1 during intoxication and ten days into abstinence, suggesting persistence of ethanol-induced upregulation of microglial genes. Altogether, these findings indicate that repetitive binge-like ethanol is sufficient to elicit changes in microglial reactivity. This altered neuroimmune state may contribute to the development of alcohol use disorders.

## 1. Introduction

Excessive alcohol consumption has been linked to many social and health consequences, including the development of alcohol use disorders (AUDs) [1, 2]. The term AUDs is an umbrella term that includes a variety of problematic drinking behaviors like alcohol dependence, repetitive binge drinking, and any ethanol consumption by at-risk populations (e.g., pregnant women or underage individuals). However, most of the societal problems associated with AUDs occur due to binge drinking [3]. Binge drinking is a form of heavy ethanol consumption that results in blood ethanol concentrations (BECs) greater than 80 mg/dL [4]. This is normally achieved with 4–5 drinks within two hours. Many theorize that repetitive binge drinking fundamentally changes normal neurobiological functions altering the pharmacological properties of alcohol and contributing to the development of alcohol dependence [5–7]. Manipulating the neuroplastic changes that occur due to binge drinking may be vital in curbing excessive alcohol consumption and reducing the propensity of AUD development. Although alcohol abuse can elicit maladaptation in various biologic systems, our work focuses on alcohol-induced microglial reactivity after repetitive binge-like alcohol consumption in the Drinking-in-the-Dark (DID) model to recapitulate human binge consumption [8].

Microglia are often called the “central nervous system macrophages” because they have a shared hematopoietic lineage with peripheral macrophages and have phagocytic activity within the brain [9]. Microglia were traditionally studied as responsive agents to infections that could mediate neurodegeneration and cell death [10]. However, the continuum of microglial activation and the corresponding variety of their homeostatic contributions to maintaining and/or restoring neuronal function has continued to rise in the literature [11, 12]. We now recognize that microglia have various functions independent of neurodegeneration in the healthy brain, including contributions to synaptic pruning [13] and constant surveillance of micro-environmental fluctuations [14, 15]. In the AUD field, most studies examining the microglial response to alcohol have focused on models of alcohol dependence [16, 17] and/or alcohol-induced neurodegeneration [18–21]. However, most people who binge drink neither experience dependence nor have evidence of neurodegeneration [22]. Determining the unique maladaptation from binge drinking is necessary as they are often distinct from those observed in dependence [23, 24]. For example, many postulate that the drive to consume alcohol switches from positive to negative reinforcement as individuals become more dependent on alcohol [25, 26]. Similarly changes in the microglial response may initially alter one function in the central nervous system during binge drinking but engage in more proinflammatory actions during dependence. Understanding the effects of alcohol on microglia under different types/stages of an AUD is crucial to determine the role of microglia within alcohol misuse and how microglia contribute to alcohol addiction.

The DID model is uniquely suited to capture the window of binge-like alcohol consumption before dependence-like phenotypes and without evidence of alcohol-induced neurodegeneration [8, 27]. For example, following multiple weeks of ethanol exposure in the DID model, it has been reported that there are no indices of withdrawal or other phenotypes associated with alcohol dependence [28, 29]. Using a self-administration model of binge-like consumption, this rodent model repeatedly achieves BECs comparable to those observed in human binge drinkers [30, 31]. The term “binge-like” is used in the manuscript when referring to animal ethanol consumption considering the limitations inherent in preclinical studies to truly reflect the multi-faceted conditions of binge drinking in humans [32, 33]. This work extends our previous findings on alcohol-induced upregulation of a proinflammatory cytokine milieu in the amygdala and hippocampus following acute binge-like consumption by determining the impact of alcohol exposure in the DID model on microglia [34, 35]. The hippocampus and amygdala have been chosen as the focal points because they are integral parts of the neurocircuitry that mediate binge drinking [36, 37].

## 2. Materials and methods

### 2.1 Animals

Male C57BL/6J mice (Jackson Laboratory; Bar Harbor, ME, USA) were individually housed in a reversed 12:12 hour light: dark cycle vivarium maintained at 22 °C. During experiments, animals had ad libitum access to water and Teklad Diet^®^ 7912X (Harlan Laboratories Inc.; Indianapolis, IN, USA) unless otherwise indicated [38]. All animals were ordered at seven weeks old and given at least a week to acclimate to the environment before starting experimentation at eight weeks. These procedures were all approved by both High Point University and North Carolina Central’s Institutional Animal Care and Use Committees following the Guidelines for the Care and Use of Laboratory Animals [39].

### 2.2 “Drinking in the Dark” procedures

**Fig. 1.**
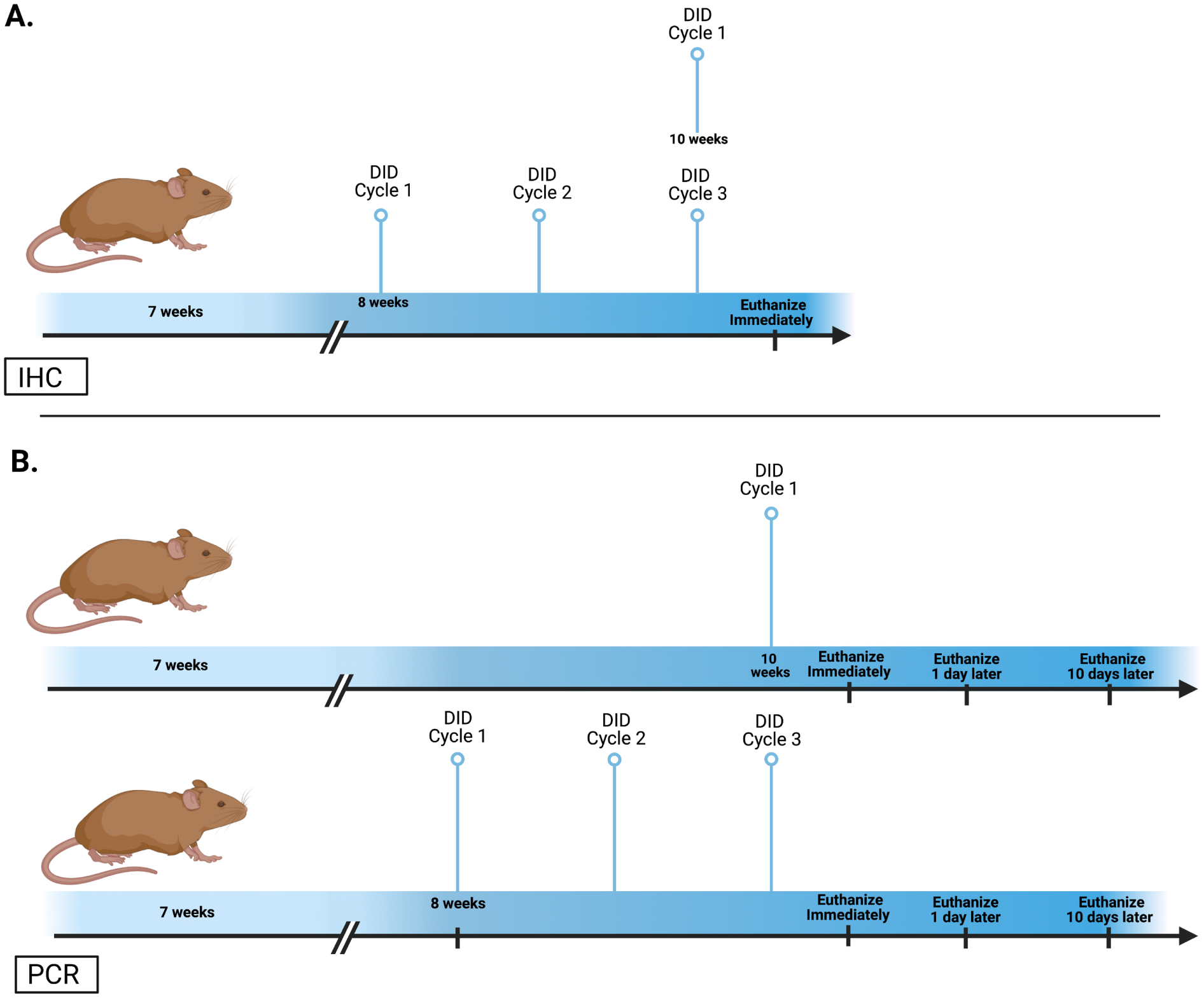
Timeline of experimental procedures. C57BL/6J mice were used for either immunohistochemistry (A) or PCR (B) after ethanol. In the IHC, mice were given ethanol, water, or sucrose for 1 or 3 DID cycles and were euthanized immediately following their last dose. However, mice only had access to ethanol or water during the DID for PCR. Moreover, ethanol animals were further subdivided into 3 groups euthanized immediately or following 1 or 10 days of abstinence.

Binge drinking was modeled using a 4-day DID paradigm as previously described [27, 28]. Briefly, home cage water bottles were removed three hours into the dark cycle. Mice received access to a single ball-bearing volumetric bottle of 20% (v/v) ethanol solution or a control solution of either 3% (w/v) sucrose or water; the presence of the ball-bearings limits leakage during administration. For the first three days of each cycle, mice had two hours of access, but on the fourth-day consumption (g/kg) was measured over four hours. Mice went through the DID procedure for 1 (H_2_O, n = 5; Sucrose, n = 8; Ethanol n = 10) or 3 cycles (H_2_O, n = 5; Sucrose, n = 9; Ethanol, n = 7) with each 4-day DID cycle separated by three days. In the PCR studies, mice underwent 1 or 3 cycles with water (n = 8/group) or 1 or 3 cycles with ethanol (N =61). The ethanol animals were further split into six groups that were euthanized immediately (1 cycle, n = 10; 3 cycle, n = 10), 1 day (1 cycle, n = 10; 3 cycle, n = 10), or 10 days (1 cycle, n = 11; 3 cycle, n = 10), after ethanol exposure [40]. For more details, see the methodology schematic in Fig. 1.

Tail blood samples (≈60 *μ*L) were taken immediately following the final DID procedure to determine BECs. In the qRT-PCR studies, blood samples were not taken in the mice euthanized after 1 or 10 days because previous studies indicate that restraint stress can impact neuroimmune genes. However, for animals euthanized immediately after the DID procedure, trunk blood was taken. Serum was obtained through centrifugation and run-in duplicates using the EnzyChrom^™^ Ethanol Assay Kit (BioAssay Systems; Hay-ward, CA, USA) [41–43].

### 2.3 Immunohistochemistry & immunoreactivity quantification

IHC was conducted to characterize the alcohol-induced microglial response within the amygdala and hippocampus. Following anesthetization with IP administration (0.1 mL) of a ketamine/xylazine cocktail (66.7 mg/mL; 6.7 mg/mL), mice used in IHC experiments were euthanized immediately after 1 or 3 cycles of DID by transcardial perfusion(0.1 M phosphate buffer saline (PBS) and 4% paraformaldehyde in PBS, pH = 7.4). The microglia-specific antibody against Iba-1 was similar to previously published studies [18, 19, 44]. Briefly, every fourth section was rinsed in a 0.1M PBS (pH = 7.4) three times for 5-minutes before a 30-minute incubation in 0.6% H_2_O_2_. After additional PBS washes, the sections were blocked using goat serum (3% goat serum/0.1% triton-X/PBS) and incubated in the rabbit anti-Iba-1 primary antibody (Wako, Richmond, VA, USA; 1:1000) or rat-anti-Ox-42 (Invitrogen, Camarillo, CA, USA; 1:500) for 48 hours at 4 °C. The primary antibody was removed with a series of PBS washes before incubation with goat anti-rabbit or goat-anti-rat secondary (1:2000; Vector Labs; Burlingame, CA, USA) for an hour. To amplify and visualize the antibody, a conjugate of avidin-biotin-peroxidase complex (ABC elite kit, Vector Labs) and the chromagen 3,3’-diaminobenzidine tetrahydrochloride (Poly-sciences; Warrington, PA, USA) was formed. Processed sections were mounted onto glass slides and coverslipped with SHUR/Mount^™^ (Triangle Biomedical Sciences; Durham, NC, USA).

Images of the hippocampus and amygdala were captured with a 10× objective using an Olympus DP73 (Olympus, Center Valley, PA, USA) attached to a Nikon Eclipse 80i microscope (Iba-1) or a B120 microscope (AmScope, Irvine, CA, USA) with an attached digital camera (MU500; Am-Scope, Ox-42). For all images, experimental bias was avoided by coding the image file before quantification. The subregions of the hippocampus (DG, CA1, and CA2/3; between Bregma −1.06 mm and −2.80 mm) and amygdala (BLA and CeA; between Bregma −0.58 mm and −2.36 mm) were individually traced [45]. Immunoreactivity was measured with the open-source software Qupath 0.2.3 (University of Edinburg, Edinburg, UK) using experimenter-determined optical density thresholds [46]. Immunoreactivity is expressed as percent area (immunoreactive positive area/total area of ROI) [47]. Microglia were also assessed using profile cell counts of Iba-1 and are expressed as Iba-1+ cells/section [18].

### 2.4 qRT-PCR

Quantitative polymerase chain reaction (qRT-PCR) was performed similarly to our previous report [47]. Briefly, un-anesthetized mice were euthanized by rapid decapitation, and the hippocampus and amygdala were microdis-sected out and snap-frozen. RNA was extracted from homogenized hippocampal or amygdala tissue using TRIzol^™^ reagent (200 *μ*L; Invitrogen). RNA concentration was normalized to 1.5 *μ*g/*μ*L following analysis with a Qubit^®^ 3.0 Fluorometer (Invitrogen). Samples were then transcribed to cDNA Synthesis using Maxima^™^ H Minus cDNA (Thermo-Scientific; Waltham MA). TaqMan^®^ assays (ThermoScientific) were used to determine the relative mRNA expression of Aif-1(Mm00479862_g1) and Itgam (Mm00434455_m1) compared with an internal control PPIA (Mm02342430_g1). Itgam (Integrin Subunit Alpha M), also known as Cd11b, is upregulated in proinflammatory microglial activation, whereas Aif-1 is associated with all microglia [48]. Measurements were compared to the water groups using the ddCT method and are expressed as the fold change, similar to previous reports [47, 49].

### 2.5 Statistical analysis

Data were analyzed and graphed using GraphPad Prism Version 7 (GraphPad Software, Inc. La Jolla, CA, USA). ANOVAs were used to assess consumption, BECs, immunoreactivity, and cell counts. Posthoc Bonferroni tests were only done if a significant interaction or main effect of ethanol was found. All data are reported as the mean ± standard error and considered significantly different if *p* < 0.05, two-tailed.

## 3. Results

### 3.1 Ethanol consumption consistent across groups

Binge-like drinking was not significantly different among the groups used in these experiments (see Table 1). The ethanol consumption data for qRT-PCR animals were collapsed across different time points (e.g., 0-day and 10-day), but BECs only represent animals in the 0-day or primary time point (Table 1). Previous studies have suggested that restraint necessary for BEC measurements may confound neuroimmune messenger ribonucleic acid (mRNA) expression due to stress [40]. Two-way analysis of variances (ANOVAs; Experimental cohort × Number of DID cycles) indicated no interaction or main effects of the experimental cohort (qRT-PCR vs. IHC) or number of cylce on either ethanol or water consumption. Importantly, the average BEC (*μ* = 117. ± 38.8) for ethanol-treated animals after four hours of consumption was above the threshold of binge-like consumption. A two-way ANOVA (Experimental cohort × Number of DID cycles) did not indicate that the BECs were significantly different between the groups.

### 3.2 Binge-like consumption has regionally specific Iba-1 immunoreactivity effects

**Fig. 2.**
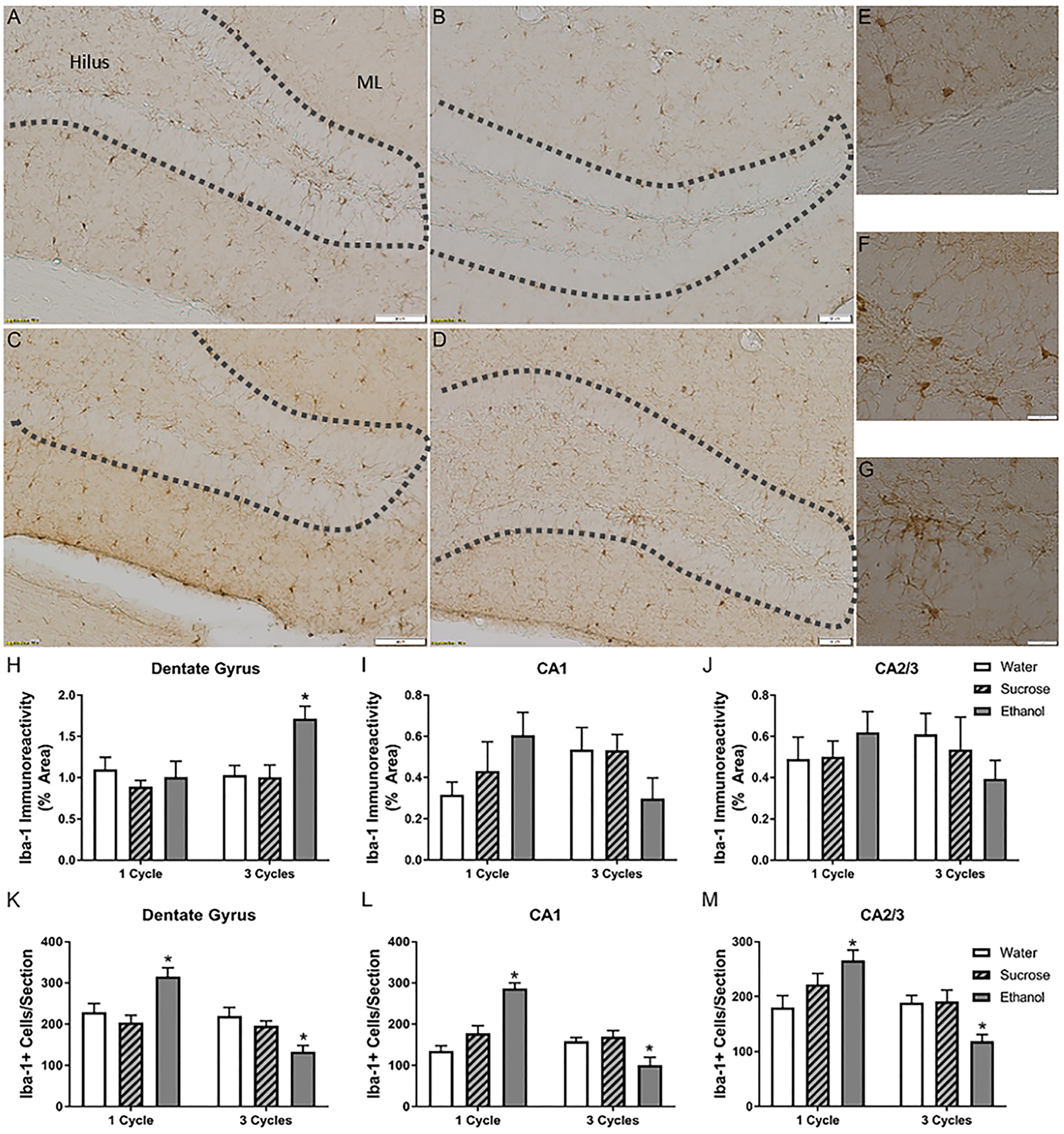
Binge-like consumption changes hippocampal microglia. Iba-1 immunoreactivity was upregulated in the dentate gyrus. Representative photomicrographs of the dentate gyrus of mice given access to 3 cycles of water (A), 3 cycles of sucrose (B), 1 cycle of EtOH (C), or 3 cycles EtOH (D). The dashed grey lines outline the edges of the granular cell layer of the DG. Insets of microglia after water (E), 1 cycle of EtOH (F), and 3 cycles of EtOH (G) highlight morphological changes in the microglia-induced binge. Quantification of the immunoreactivity (IR) indicated that after 3 cycles of EtOH, there was an increase in the IR in the DG (H) but not the cornu amonis regions (I, J). Cell counts indicated that the number of microglia in the hippocampus increased after ethanol exposure but decreased after 3 cycles (K–M). *p > 0.05 compared to water; Scale bars in representative photomicrographs = 50 *μ*m. ML, molecular layer.

Microglial activation results in morphological changes, which can be assessed using densitometric analysis of immunohistochemical markers like ionized calcium-binding adaptor 1 (Iba-1) [19, 48]. Two-way ANOVAs (Treatment × Number of Cycles) indicated that there was no interaction or main effects of treatment or number of DID cycles on Iba-1 density in the cornu amonis (CA) 1 or CA2/3 regions; however, in the dentate gyrus (DG), an interaction [F_(2,43)_ = 3.57, *p* = 0.037] and the main effect of treatment [F_(2,43)_ = 4.68, *p* = 0.015] but no main effect of number of cycles was observed. Posthoc analyses indicated a significant increase in immunoreactivity in the ethanol group after three cycles (Fig. 2H).

Because immunoreactivity can also be changed by the number of microglia and not just microglial morphological variations, Iba-1+ cells were also counted. The number of microglia seemed to be more sensitive to alcohol exposure than immunoreactivity. Two way ANOVAs (Treatment × Number of Cycles) indicated an interaction between number of cycles and treatment in the DG [F_(2,43)_ = 16.46, *p* < 0.0001], CA1 [F_(2,43)_ = 21.81, *p* < 0.0001], and in the CA2/3 fields [F_(2,43)_ = 8.53, *p* < 0.001]. Posthoc Dunnet’s tests indicated an increase in the number of microglia in the DG, CA1, and CA2/3 regions after one cycle of ethanol; however, a decrease after three cycles was observed among ethanol animals in each of the hippocampal subregions (Fig. 2K–M). After ethanol exposure (Fig. 2F, G), photomicrographs show that hippocampal Iba-1+ cells appear to have a more activated morphology than water controls (Fig. 2E). The immunoreactivity per cell was calculated (Table 2). Two-way ANOVAs (Treatment × Number of Cycles) indicated no interaction or main effects on immunoreactivity per cell in either the CA2/3 or the CA1, but in the DG, there was a main effect of ethanol [F_(2,43)_ = 14.56, *p* < 0.0001]. Post-hoc Dunnet’s test indicated that after 1 and 3 cycles of ethanol, there was a significant increase in the immunoreactivity/cell compared with the water control group.

**Fig. 3.**
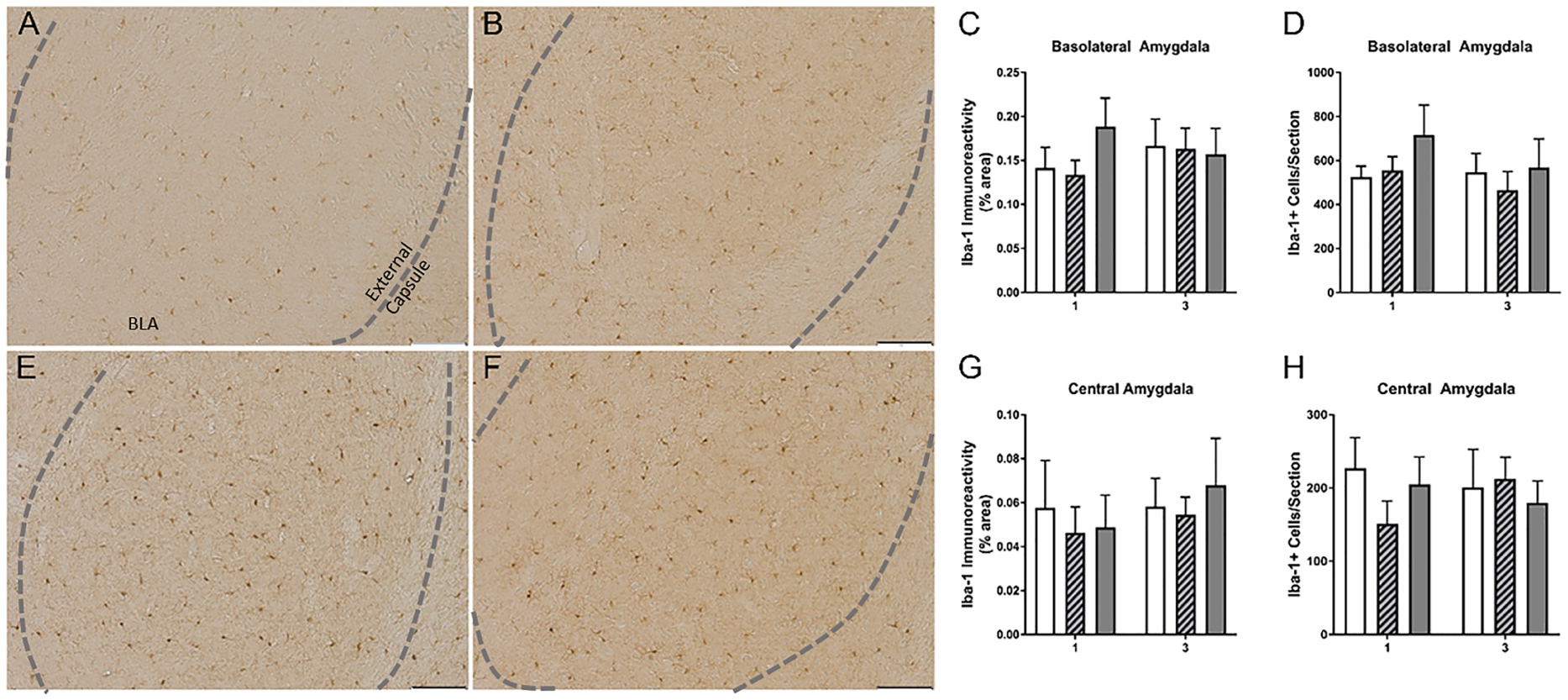
Binge-like consumption did not significantly impact the microglia of the amygdala. Representative photomicrographs of microglia from the basolateral amygdala (BLA) indicate consistent density and morphology in mice with 3 cycles of water (A), 3 cycles of sucrose (B), 1 cycle of EtOH (E), or 3 cycles EtOH (F). The dashed grey lines outline the ventral boundaries of the external capsule along the BLA. Quantification of the immunoreactivity (IR) indicated that EtOH did not affect the IR in either BLA (C) or the CeA (G). Likewise, no significant differences were observed in the microglia number (D, H). Scale bars in representative photomicrographs = 50 *μ*m.

There was no effect of ethanol on the microglia of the amygdala. No interaction or main effects of either treatment or number of cycles was observed on Iba-1 immunoreactivity in the basolateral amygdala (BLA) or central amygdala (CeA; Fig. 3). Likewise, a two-way ANOVA indicated no significant influence of ethanol on the microglia number in the BLA or CeA (Fig. 3).

### 3.3 Increased CR3 expression in microglia after binge-like consumption

**Fig. 4.**
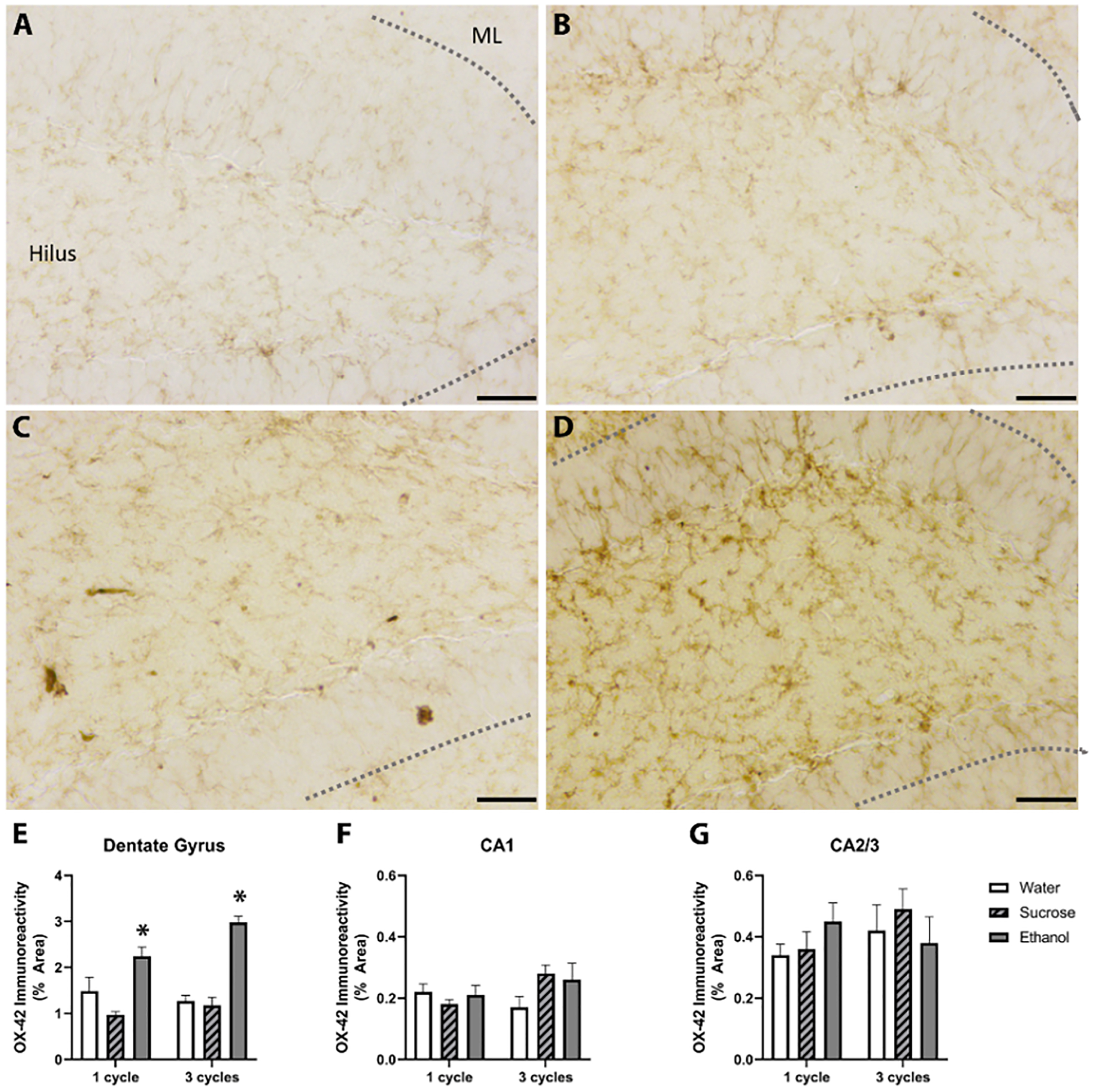
Binge-like consumption increases microglial expression of the immunologic CR3. Ox-42 immunoreactivity was upregulated in the dentate gyrus. Representative photomicrographs of the dentate gyrus of mice given access to 3 cycles of water (A), 3 cycles of sucrose (B), 1 cycle of EtOH (C), or 3 cycles of EtOH (D). The dashed grey lines outline the edges of the granular cell layer of the DG. Quantification of the immunoreactivity (IR) indicated that after 1 and 3 cycles of EtOH, there were increases in the IR in the DG (E) but not the cornu amonis regions (F, G). *p > 0.05 compared to water; Scale bars in representative photomicrographs = 75 *μ*m. ML, molecular layer.

Iba-1 is a cytoskeletal protein in microglia whose upregulation suggests morphological changes or increased numbers. However, Ox-42 recognizes the transmembrane complement receptor 3. Its upregulation is purported to indicate increased microglial adhesion to damaged cells and pathogens [19, 50]. Two-way ANOVAs (Treatment × Number of Cycles) indicated that there was no interaction or main effects of treatment or number of DID cycles on Ox-42 immunoreactivity in the CA1 or CA2/3 regions; however, in the DG an interaction [F_(2,43)_= 3.58, *p* = 0.037] and main effect of treatment [F_(2,43)_= 53.6, *p* < 0.0001] were observed without a no main effect of number of cycles was observed. Posthoc analyses indicated a significant increase in immunoreactivity in the ethanol group after both one and three cycles of ethanol (Fig. 4E).

### 3.4 Repeated binge-Like consumption causes persisting changes in microglial mRNA

**Fig. 5.**
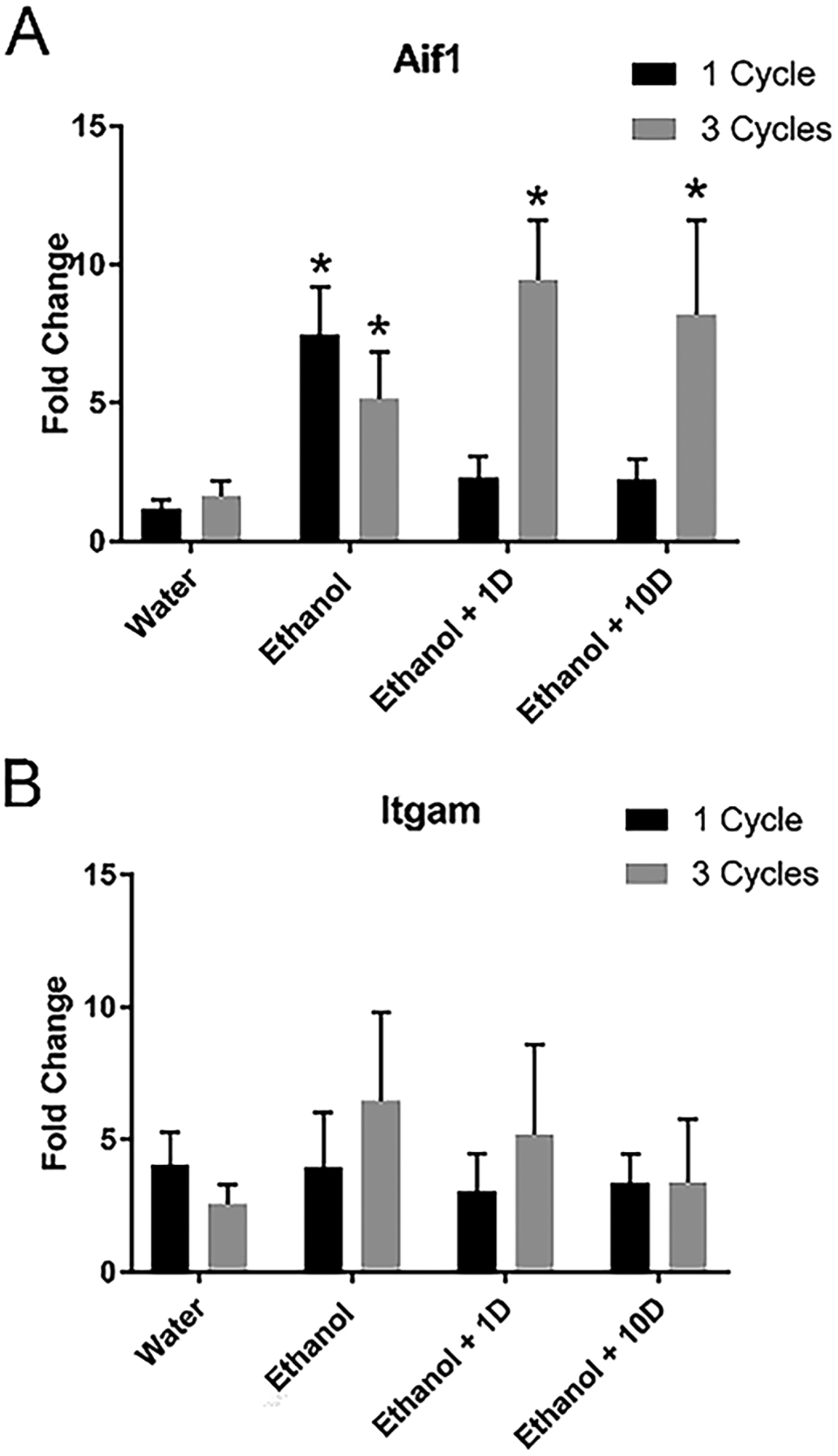
Ethanol increases microglial mRNA. After both 1 and 3 weeks of ethanol, Aif-1 was significantly upregulated, but only after 3 weeks of ethanol exposure did the increase persist even 10 days after binge-like ethanol consumption (A). No significant changes were observed in Itgam expression (B). *p > 0.05 compared to water.

To further explore the changes in microglial reactivity to binge-like consumption, qRT-PCR was used to determine if changes persisted into abstinence. A two-way ANOVA (Treatment group × Number of Cycles) indicated that there was no interaction or main effects of ethanol or time on Itgam expression (Fig. 5B); however, an analysis of Aif-1 mRNA indicated there was an interaction [F_(3,69)_= 3.39, *p* = 0.023] and main effect of the number of cycles [F_(1,69)_= 4.79, *p* =0.032]. Posthoc Dunnet’s test indicated an increase in Aif-1 expression during intoxication after both 1 and 3-DID cycles; however, only after three cycles of ethanol was a significant increase in Aif-1 expression observed during abstinence (Fig. 5A).

## 4. Discussion

Our previous studies have examined the effects of repetitive binge-like consumption on cytokines and astrocytes [34, 35, 47]. However, this manuscript extends those studies by examining the impact of ethanol consumption in the DID model on microglia. Given the critical role of microglia in modulating psycho-neuroimmune and neuroinflammatory responses [51, 52], highlighting the specific effects of binge-like alcohol consumption on microglia is critical to fully understanding the neuroimmune response associated with excessive alcohol use. The major findings of this paper are that: (1) binge-like ethanol consumption results in changes in the number, morphology, and immunological status of microglia, (2) the hippocampus is more sensitive to alcohol-induced microglial effects than the amygdala, and (3) repeated exposure results in a persistent change in microglial gene expression.

The hippocampus has been repeatedly shown to be susceptible to ethanol abuse affecting important neurologic functions, including neurogenesis [53], memory [54, 55], and neurodegeneration [56, 57]. The interesting thing about neuroimmune dysregulation is that it can impact the afore-mentioned alcohol-induced effects in the hippocampus [53, 58]. Our results uniquely show that the DID model induces changes in microglia of the hippocampus. Immunohistochemistry analysis initially revealed that binge-like consumption increased immunoreactivity only after three cycles in the DG. Iba-1 immunoreactivity analysis is usually purported to be a sign of microglial activation [59, 60]; however, our qualitative observations of the microglial morphology led us to believe that after one cycle of ethanol exposure, microglia still appeared activated despite the overall decrease in immunoreactivity. We estimated the immunoreactivity per cell by counting the total number of cells, which we believe translates closely to the average microglial size. Our data suggest that after both 1 and 3-DID cycles of ethanol exposure, the immunoreactivity/cell increases. Changes in the morphological presentation of microglia are usually thought to indicate microglial activation. The increase in Ox-42 further supported binge-alcohol-induced microglial activation after the DID. Ox-42 upregulation indicates that the microglia are immunologically active. The differential pattern Iba-1 and Ox-42 observed may suggest that microglia populations remained primed despite less robust morphological presentations [18, 61]. Our findings concur with other studies indicating that alcohol can induce increases in Iba-1 and Ox-42 immunoreactivity, including models of alcohol dependence [62–64], alcohol-induced neurodegeneration [18, 21], and fetal alcohol spectrum disorders [65, 66], but our results have uniquely shown the effects of alcohol before dependence and at much lower BECs.

We also observed a change in microglia number after the DID paradigm. After one cycle of ethanol, there was an increase in microglia throughout the hippocampus. Alcohol-dependent models with higher BECs have reported that microglia begin to proliferate after a period of alcohol abstinence [44, 67], but other rodent models with BECs below binge levels did not observe proliferating microglia even after 7 weeks [68]. Together, these data suggest something unique occurs after binge-like consumption that initially increases the number of microglia compared with other alcohol exposure models. We did not determine whether the increase in microglia was due to a migration of microglia to the hippocampus or microglial cell proliferation. After additional ethanol exposure, our data indicated a significant decrease in the number of microglia in the hippocampus. We did not directly elucidate whether microglia were damaged by repeated exposure, but previous reports have also seen decreases in microglia number in alcohol-related brain damage models [18, 69]. It is important to denote that microglia proliferation and loss are sex-dependent [69, 70], and we only focused on male mice. A decrease in microglia number as alcohol exposure increases may align with the human condition. Imaging studies of alcoholics have shown that a down-regulation in the microglial markers for translocator protein (TSPO) correlates with the dependence severity [71, 72]. An important caveat to consider is that a recent paper [73] observed opposing effects between *in vivo* imaging and autoradiography using TSPO markers. The loss discovered after just three weeks of repetitive binge-like consumption in a model of non-dependence may be the precursor to the glial changes that occur during alcohol-dependence.

Our studies found no effects of DID ethanol consumption on the microglia of the amygdala. This was surprising given that microglia are the initial responders in a neuroimmune response. We have previously found that binge-like consumption is associated with a more proinflammatory cytokine environment in the BLA [34, 35]. Because the hippocampus and amygdala are both susceptible to alcohol-induced neuroplasticity, we expected similar findings between the hippocampus and the amygdala [73, 74]. However, the differences observed herein between the regions may indicate that withdrawal and/or chronic ethanol exposure is integral for changes in the amygdala. Our data only looked at microglia during intoxication in the amygdala. It is also possible that the lack of changes in the amygdala may be associated with the lower density of Iba-1 in the region compared with the hippocampus. A recent meta-analysis attempted to determine the relative density of microglia in the mouse brain. Still, there was insufficient evidence for any strong conclusions concerning microglia in the amygdala compared with other regions [75]. However, in the hippocampus, the DG and CA1 regions, where the microglia appear to be most susceptible to alcohol, have a greater density of microglial cells than the CA2/3 region [75, 76]. Moreover, in pathologies like aging, hippocampal microglia appear more reactive than other brain regions [77]. Our recent work suggests that excessive alcohol intake may interact with aging cascades to dysregulate microglia [43]. Whether the lack of effect observed herein is due to differences in basal density or reactivity is unknown. However, our data does agree with other studies that did not find that ethanol impacted microglia in the amygdala [78]. Future studies should explore other regions correlated to alcohol abuse and control regions to determine the uniqueness of the hippocampal findings. At a minimum, our results show that hippocampal microglia are more sensitive to repetitive alcohol consumption compared with the amygdala.

To determine whether the alcohol-induced changes in microglia of the hippocampus persisted into abstinence, we elected to use qRT-PCR. After one 4-Day DID cycle of ethanol exposure, Aif-1 mRNA initially increases but appears to quickly resolve in abstinence. Conversely, the increase in Aif-1 mRNA was present during intoxication and persisted even 10 days after binge-like consumption. No changes were seen in Itgam after ethanol exposure, but this may be because the concentration of mRNA of Itgam was lower than either Aif-1 according to the average cycle threshold (CT) values of the water group. The lower expression of Itgam compared with other markers of microglia has previously been observed [79] and reinforces the hypothesis that Itgam or CD11b is a marker of more activated microglia compared with Aif-1 (also known as Iba-1) [19, 48]. Other AUD models have also reported that ethanol affects the microglial transcriptome, including research examining the prefrontal cortex in the DID model [17, 80]. We, however, uniquely examined the impact of repetitive binge drinking in the hippocampus. Together this information may indicate that binge-like consumption drives changes in microglia that are not fully activated or proinflammatory at BECs that are often observed in non-dependent, problematic alcohol drinkers [81]. Previous research agrees with these findings that as ethanol exposure increases, it drives the microglial response to a more pro-inflammatory state [18, 82], especially as it relates to the microglial transcriptome [17]. Furthermore, these findings concur with other research showing that acute ethanol only causes transient microglial morphology changes compared with more chronic use [83].

## 5. Conclusions

The impact of binge-like consumption on neuroinflammation continues to be of interest. Alcohol-induced neuroinflammatory signaling can underlie both the behavioral and biological consequences observed in the development of AUDs. Our data reveal how binge drinking impacts the microglial response before dependence without significant neurodegeneration. Given that the BECs elicited by this model (~100 mg/dL) is much lower than the previous studies of alcohol-related brain damage (~300 mg/dL) that showed microglial activation, it could indicate that repetitive binge consumption is problematic, rather than dependent individuals, may lead to maladaptations in the glial responses. Because alcohol abuse is comorbid with so many other conditions that affect the neuroimmune response, including aging, infections, and traumatic brain injury, these data provide a snapshot to consider how binge-like consumption’s alteration of microglia may contribute to the worsening of neuroinflammatory events and promote further alcohol misuse.

## Figures and Tables

**Table 1.** Drinking in the Dark Binge Data.

Study	Group	Consumption (g/kg)		BEC (mg/dL)	
		1 week	3 week	1 week	3 week
IHC	Ethanol	5.0 ± 0.4 (n = 9)	4.7 ± 0.4 (n = 10)	114.1 ± 10.4	122.4 ± 19.2
	Sucrose	5.5 ± 0.4 (n = 10)	5.7 ± 0.5 (n = 10)	-	-
	Water	66.5 ± 2.8 (n = 5)	70.0 ± 4.7 (n = 5)	-	-
PCR	Ethanol	5.4 ± 0.3 (n = 16)	4.9 ± 0.2 (n = 16)	102.3 ± 21.3	129.9 ± 15.5
	Water	70.4 ± 3.5 (n = 6)	57.0 ± 6.7 (n = 6)	-	-

**Table 2.** Immunoreactivity per cell.

Brain Region	Water		Sucrose		Ethanol	
	1 cycle	3 cycles	1 cycle	3 cycles	1 cycle	3 cycles
CA1	0.002 ± 0.0006	0.003 ± 0.0007	0.002 ± 0.0011	0.003 ± 4.6e-4	0.002 ± 0.0003	0.004 ± 0.0017
CA2/3	0.003 ± 0.0007	0.003 ± 0.0005	0.003 ± 0.0006	0.003 ± 0.0011	0.002 ± 0.0005	0.004 ± 0.0009
DG	0.005 ± 0.0013	0.005 ± 0.0010	0.005 ± 0.0007	0.005 ± 0.0007	0.011 ± 0.0023*	0.015 ± 0.0025*

* *p* > 0.001 water compared with ethanol.

## Abbreviations

AUDs: Alcohol Use Disorders
Aif-1: Allograft Inflammatory Factor 1
ANOVAs: Analysis of Variances
BLA: Basolateral Amygdala
BECs: Blood Ethanol Concentrations
CeA: Central Amygdala
CA: Cornu Amonis
CT: Cycle Threshold
DG: Dentate Gyrus
DID: Drinking-in-the-Dark
IHC: Immunohistochemistry
ITGAM: Integrin alpha M
Iba-1: Ionized Calcium Binding Adaptor 1
mRNA: Messenger Ribonucleic Acid
PBS: Phosphate Buffer Saline
TSPO: Quantitative Reverse Transcription; Translocator Protein
